# Attenuated Notch signaling in schizophrenia and bipolar disorder

**DOI:** 10.1038/s41598-018-23703-w

**Published:** 2018-03-28

**Authors:** Eva Z. Hoseth, Florian Krull, Ingrid Dieset, Ragni H. Mørch, Sigrun Hope, Erlend S. Gardsjord, Nils Eiel Steen, Ingrid Melle, Hans-Richard Brattbakk, Vidar M. Steen, Pål Aukrust, Srdjan Djurovic, Ole A. Andreassen, Thor Ueland

**Affiliations:** 1 NORMENT, KG Jebsen Centre for Psychosis Research, Institute of Clinical Medicine, University of Oslo, and Division of Mental Health and Addiction, Oslo University Hospital, Oslo, Norway; 2 Division of Mental Health and Addiction, Møre and Romsdal Hospital Trust, Kristiansund, Norway; 3 Departent of Neurohabilitation, Division of Neurology, Oslo University Hospital, Oslo, Norway; 4 Research Institute for Internal Medicine, Oslo University Hospital Rikshospitalet, Oslo, Norway; 5 NORMENT, KG Jebsen Centre for Psychosis Research, Department of Clinical Science, University of Bergen, Bergen, Norway; 6 Dr. Einar Martens Research Group for Biological Psychiatry, Center for Medical Genetics and Molecular Medicine, Haukeland University Hospital, Bergen, Norway; 7 Section of Clinical Immunology and Infectious Diseases, Oslo University Hospital Rikshospitalet, Oslo, Norway; 8 Instiute of Clinical Medicine, Oslo University Hospital Rikshospitalet, Oslo, Norway; 9 K.G. Jensen inflammatory Research Center, University of Oslo, Oslo, Norway; 10 K. G. Jebsen Thrombosis Research and Expertise Center, University of Tromsø, Tromsø, Norway; 11 Department of Medical Genetics, Oslo University Hospital, Oslo, Norway; 12 NORMENT, KG Jebsen Centre for Psychosis Research, Department of Clinical Science, University of Bergen, Bergen, Norway

## Abstract

The Notch signaling pathway plays a crucial role in neurodevelopment and in adult brain homeostasis. We aimed to further investigate Notch pathway activity in bipolar disorder (BD) and schizophrenia (SCZ) by conducting a pathway analysis. We measured plasma levels of Notch ligands (DLL1 and DLK1) using enzyme immunoassays in a large sample of patients (SCZ *n* = 551, BD *n* = 246) and healthy controls (HC *n* = 639). We also determined Notch pathway related gene expression levels by microarray analyses from whole blood in a subsample (SCZ *n* = 338, BD *n* = 241 and HC *n* = 263). We found significantly elevated Notch ligand levels in plasma in both SCZ and BD compared to HC. Significant gene expression findings included increased levels of *RFNG* and *KAT2B* (*p* < 0.001), and decreased levels of *PSEN1* and *CREBBP* in both patient groups (*p* < 0.001). *RBPJ* was significantly lower in SCZ *vs* HC (*p* < 0.001), and patients using lithium had higher levels of *RBPJ* (*p* < 0.001). We provide evidence of altered Notch signaling in both SCZ and BD compared to HC, and suggest that Notch signaling pathway may be disturbed in these disorders. Lithium may ameliorate aberrant Notch signaling. We propose that drugs targeting Notch pathway could be relevant in the treatment of psychotic disorders.

## Introduction

Schizophrenia (SCZ) and bipolar disorder (BD) are severe mental disorders that have prompted extensive research as they are among the leading causes of worldwide disability^1,2^. The neurodevelopmental hypothesis for SCZ suggests that the neuroanatomical defects associated with SCZ are caused by dysregulation of brain development^3^. In BD, brain morphological alterations are more subtle, but these together with behavioral changes prior to onset of illness support neurodevelopmental abnormalities also for BD^4^. In addition to developmental abnormalities, accelerated grey matter decline, aberrant brain connectivity and biochemical changes in the adult brain may indicate neurodegenerative processes in SCZ^5,6^; this may also be present in BD, albeit substantially less prominent^7^.

Notch signaling is well known as a master regulator of neural stem cells and neural development, and orchestrates nervous system development and patterning by regulating neurogenesis, axonal growth, synaptogenesis and predisposing neurons to apoptosis also in the adult brain^8^. These facts make it a pertinent candidate for exploration in psychotic disorders. The Notch signaling pathway was first associated with SCZ through genetic findings linking the *NOTCH4* gene to SCZ in British parent-offspring trios^9^, and later confirmed by larger genome wide association studies^10^. Initial studies investigating Notch in BD were inconclusive^11,12^, however, in 2012 we found increased gene expression of *NOTCH4* in BD^13^.

In addition to being important in regulating neural cell proliferation, differentiation, and neural cellular growth^8^, Notch is a crucial contributor in adaptive and innate immune responses^14^. Notch and its ligands (*e.g*. delta-like 1, DLL1) have also been implicated in endothelial cell dysregulation and vascular inflammation^15,16^ as well as macrophage activation^17^, and is involved in the interaction between immune cells and the brain during ischemic stroke. This may be relevant also in relation to severe psychotic disorders as immune-pathogenic mechanisms have been implicated in SCZ and BD, partly based on the demonstration of low-grade systemic inflammation including T cell activation in these patients^18^.

Based on the emerging significance of neuro-inflammation and immunogenetics in SCZ and BD, we hypothesized that Notch signaling components in inflammatory cells, both as a proxy for CNS tissues, but also representing important cells that could modulate different neural processes, would be dysregulated. Thus, we aimed to characterize the Notch signaling pathway in patients with SCZ, BD and healthy controls (HC) by conducting a pathway analysis on the mRNA level in whole blood, as well as investigating plasma levels of secreted Notch ligands. Our secondary aim was to investigate whether potential alterations in Notch pathway mRNA expression or secreted ligands are associated with the use of psychotropic medication.

## Results

### Demographics and clinical characteristics

Socio-demographic and clinical characteristics of the participants are shown in Table 1. The plasma protein cohort and the whole blood mRNA cohort showed the same differences between SCZ, BD and HC groups with the exception of one clinical characteristic: SCZ patients had higher CDSS scores in whole blood cohort, but not in the plasma protein cohort. The main differences between patients and HC were age, ethnicity and sex.

**Table 1:** Demographic and clinical characteristics of participants.

Parameters	Plasma (Notch ligand) cohort				Whole blood (mRNA) cohort			
	SCZ (N = 551)	BD (N = 246)	HC (N = 639)	Post Hoc Analysis	SCZ (N = 338)	BD (N = 241)	HC (N = 263)	Post Hoc Analysis
Male sex, N (%)	334 (60.6)	97 (39.4)	364 (57.0)	SCZ > HC > BD	275 (61.5)	90 (39.5)	144 (54.8)	SCZ > HC > BD
Ethnicity (Cauc. %)	444 (80.6)	213 (86.6)	629 (98.4)	HC > BD > SCZ	228 (90.1)	140 (94.6)	208 (100)	HC > BD > SCZ
***Medication N(%)***:								
Antipsychotics	510 (84.6)	167 (66.0)	—	SCZ > BD	234 (92.5)	114 (77.0)	—	SCZ > BD
Lithium	12 (2.0)	51 (20.2)	—	BD > SCZ	3 (1.2)	32 (21.6)	—	BD > SCZ
Antidepressants	179 (31.5)	95 (38.8)	—	BD > SCZ	35 (13.8)	66 (44.6)	—	BD > SCZ
Mood stabilizers	56 (9.3)	87 (34.4)	—	BD > SCZ	78 (30.8)	57 (38.5)	—	BD > SCZ
Age (years)	27 (13)	29 (18)	31 (13)	BD, HC > SCZ	29 (14)	35 (20)	31.0 (13)	BD, HC > SCZ
DOI (years)	4 (8)	4 (10)	—	BD > SCZ	4 (9)	5 (13)	—	BD > SCZ
PANSS total score	62 (22)	44 (13)	—	SCZ > BD	64 (24)	45 (14)	—	SCZ > BD
YMRS total score	3 (9)	2 (5)	—	SCZ > BD	3 (9)	2 (6)	—	SCZ > BD
IDS total score	17 (19)	17 (16)	—	NS	18 (18)	15 (16)	—	NS
CDSS total score	5 (8)	4 (6)	—	NS	5 (7)	3 (7)	—	SCZ > BD
GAF-S	40 (15)	57 (16)	—	BD > SCZ	40 (14)	54 (17)	—	BD > SCZ
GAF-F	42 (14)	51 (19)	—	BD > SCZ	42 (15)	50 (17)	—	BD > SCZ

Abbreviations: SCZ = Schizophrenia; BD = Bipolar Disorder; HC = Healthy Controls; Cauc. = Caucasians; NS = Non-Significant; DOI = Duration of illness; PANSS = Positive and Negative Syndrome Scale; YMRS = Young Mania Rating Scale; IDS = Inventory of Depressive Symptoms; CDSS = Calgary Depression Scale for Schizophrenia; GAF-S = Global Assessment of Functioning - Symptom Scale; GAF-F = Global Assessment of Functioning - Function Scale.

Categorical data are given as percent in brackets, while continuous data are given as median with interquartile range. Post hoc analysis is performed using Pearson Chi-square for categorical data, and Mann-Whitney U tests for continuous data. Differences between groups are significant when *p* < 0.05.

### Plasma levels of Notch ligands

The plasma levels of the secreted Notch ligands and group comparisons are summarized in Table 2. Patients had significantly higher plasma levels of DLL1 compared to HCs, with SCZ having significantly higher levels than BD after controlling for age and gender. DLK1 levels showed nominally significant increase in both patient groups compared to HC with no differences between BD and SCZ.

**Table 2:** Differences between groups for plasma markers of Notch pathway after controlling for age and gender.

plasma ligands	*M(IQR)*			SCZ vs. HC			BD vs. HC			SCZ vs. BD		
	SCZ	BD	HC	*df*	*t*	*F*	*df*	*t*	*F*	*df*	*t*	*F*
DLL1	5 (1.8)	4.7 (1.6)	4.5 (1.3)	1232	8.8***	32.01***	889	3.26**	10.81***	848	3.00**	9.13***
DLK1	186 (170)	188 (164)	180 (149)	1243	2.23*	10.62***	893	2.00*	11.38***	854	0.08	9.06***

*p < 0.05 **p < 0.03 ***p < 0.001

Abbreviations: M = median; IQR = interquartile range; SCZ = schizophrenia; BD = bipolar disorder; HC = healthy controls; DLL1 = Delta-like protein 1; DLK1 = Delta Like Non-Canonical Notch Ligand 1.

ANCOVA using linear regression models. Results are significant if *p* < 0.03, and nominally significant if 0.03 < *p* < 0.05 (Bonferroni correction).

### Gene expression in whole blood

**Figure 1:**
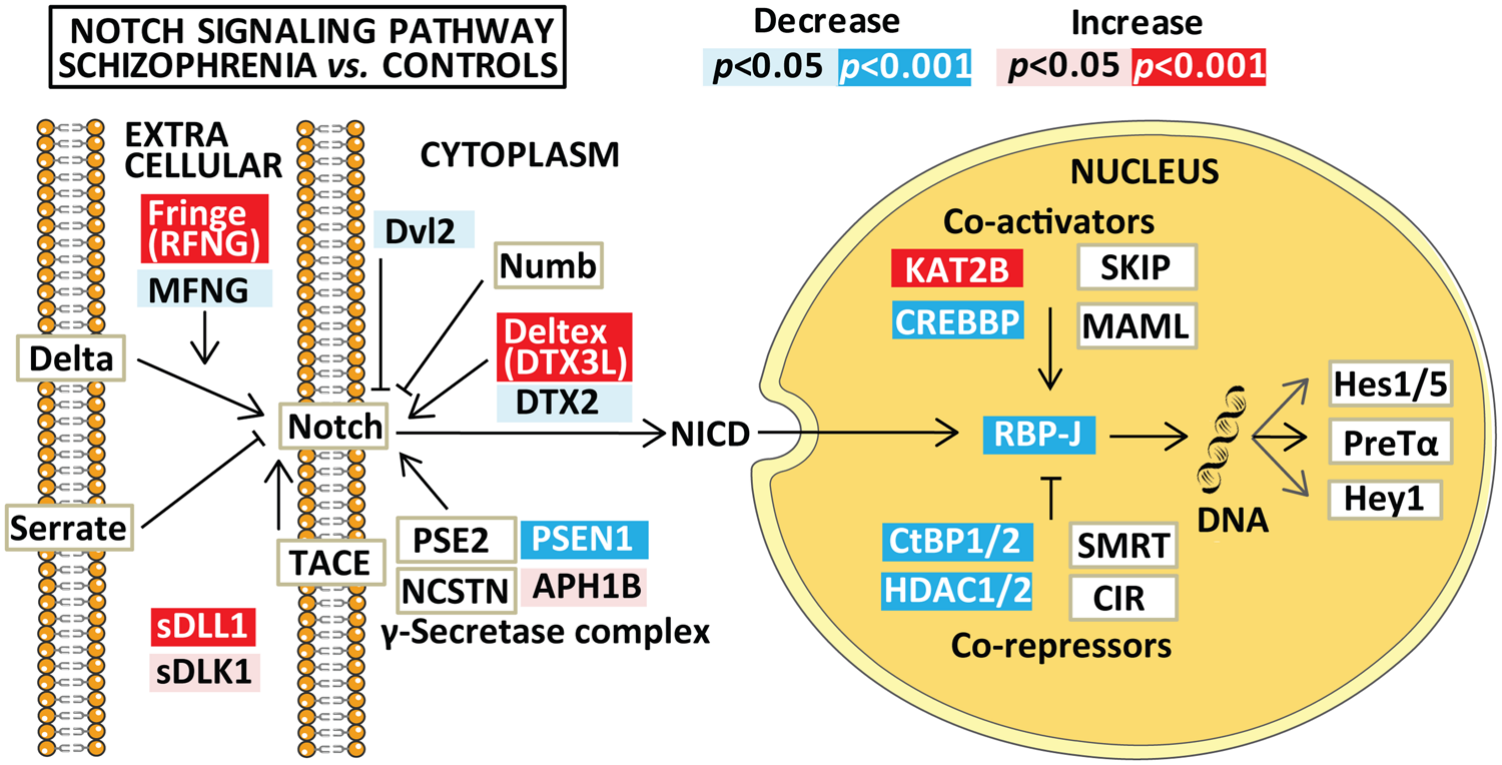
Summary of significant and nominally significant findings in Notch pathway mRNA expression between the schizophrenia and the healthy control group after controlling for age, gender and *Bmal1*. Results are given as *p*-values, adjusted for multiple testing, where significant results are indicated in red/dark blue (for increased/decreased mRNA expression) and nominally significant results (0.001 < *p* < 0.05) are shown as pink/light blue (for increased/decreased mRNA expression). Non-significant results are depicted as boxes with white background. The figure is based on the Notch signaling pathway in the KEGG database (hsa04330, version date 5/9/17). Hey1 is included by the authors due to its known role as a target gene for Notch signaling^30^.

**Figure 2:**
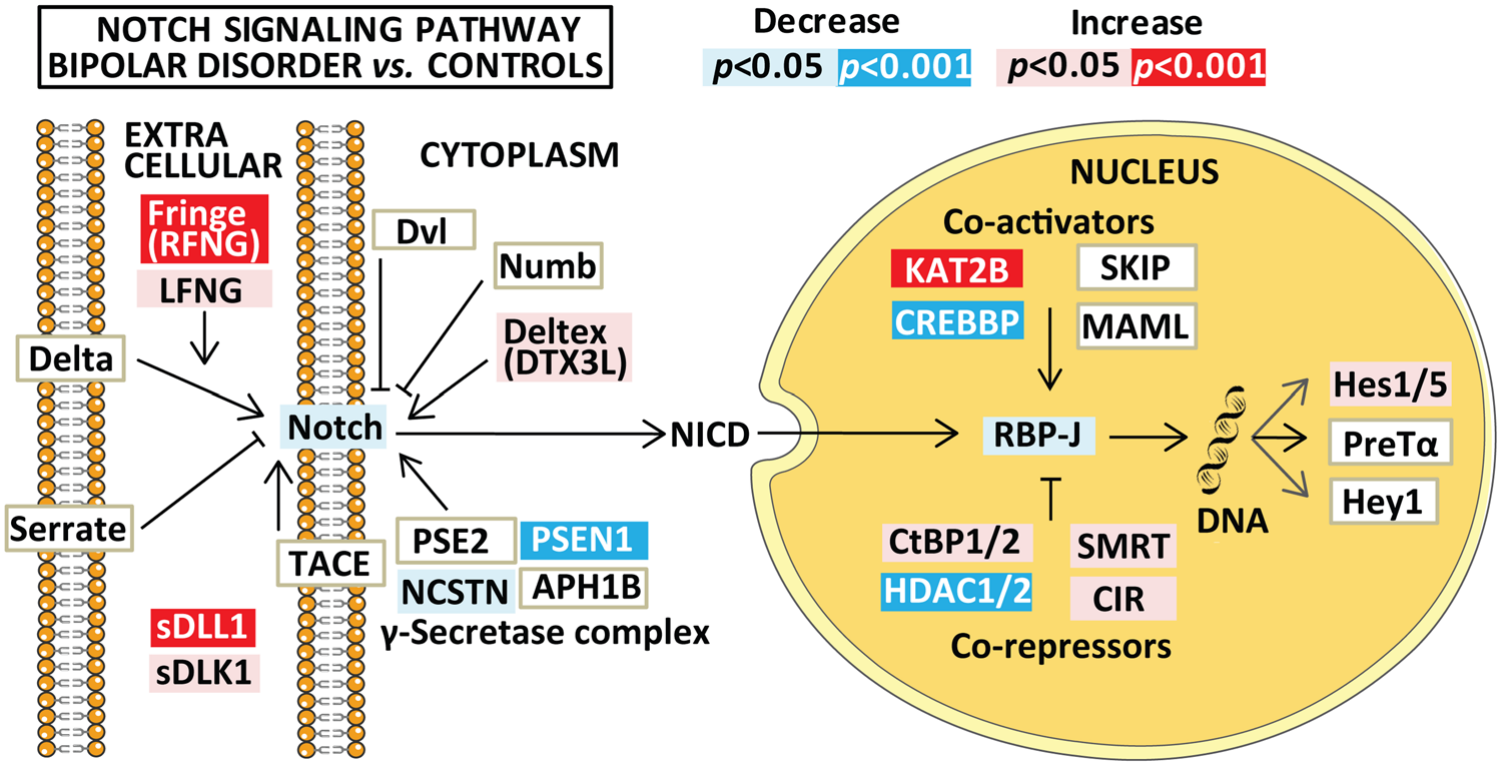
Summary of significant and nominally significant findings in Notch pathway mRNA expression between the bipolar disorder and the healthy control group after controlling for age, gender and *Bmal1*. Results are given as *p*-values, adjusted for multiple testing, where significant results are indicated in red/dark blue (for increased/decreased mRNA expression) and nominally significant results (0.001 < *p* < 0.05) are shown as pink/light blue (for increased/decreased mRNA expression). Non-significant results are depicted as boxes with white background. The figure is based on the Notch signaling pathway in the KEGG database (hsa04330, version date 5/9/17). Hey1 is included by the authors due to its known role as a target gene for Notch signaling^30^.

The mRNA expression of Notch pathway genes are summarized in Figs 1 and 2 and Table 3, and nominally significant findings (0.001 < *p* < 0.05) in Supplementary Table 1. Effect size estimates were small in general with the highest value on 0.16 for *KAT2B*. Compared to HC, both SCZ and BD had increased levels of *RFNG* and *KAT2B*, and decreased levels of *PSEN1* and *CREBBP* mRNA expression. In addition, the SCZ group had increased *DTX3L* and decreased *RBPJ, CTBP* and *HDAC* mRNA expression compared to HC (Fig. 1). The main significant differences between SCZ and BD were lower *LFNG* and increased *NOTCH2* in SCZ *vs* BD (Table 3 and Supplementary Figure 1).

**Table 3:** Significant differences between patients and controls in Notch signaling pathway gene mRNA expression after controlling for age and gender.

	Genes	specificity	SCZ vs. HC	BD vs. HC	SCZ vs. BD
			*B*	*B*	*B*
*Receptor and Cytoplasm*	*LFNG*	+++	−0.01	0.07**	−0.09***
	*RFNG*	+++	0.11***	0.07***	0.03
	*NOTCH2*	+++	0.01	−0.04**	0.06***
	*DTX3L*	+++	0.05***	0.00	0.05**
	*PSEN1*	++	−0.03***	−0.02***	−0.01
*Nucleus*	*KAT2B*	++	0.16***	0.13***	0.03
	*CREBBP*	+	−0.10***	−0.09***	−0.01
	*CTBP1*	++	−0.03***	−0.01	−0.02*
	*CTBP2*	++	−0.03***	−0.01	−0.02**
	*HDAC1*	++	−0.08***	−0.04*	−0.04*
	*HDAC2*	++	−0.06***	−0.05*	−0.01
	*RBPJ*	+	−0.07***	−0.01	−0.06**

*p < 0.05 **p < 0.01 ***p < 0.001

Specificity: + (unspecific, involved in many pathways), ++ (involved in up to 3 additional pathways *e.g*. Wnt, NF-kappa), +++ (exclusively Notch pathway related gene).

Abbreviations: SCZ = Schizophrenia; BD = Bipolar disorder; HC = Healthy controls; B = Unstandardized regression coefficient.

Gene names are listed according to the HUGO Gene Nomenclature Committee.

Results are given as effect size estimates from the linear regression analysis after correction for age, sex and *BMAL1* expression. Results are significant if *p* < 0.001, and nominally significant if 0.001 < *p* < 0.05 (Bonferroni correction).

### Role of medication

We found that patients using lithium (n = 35) had significantly higher *RBPJ* expression compared to patients not taking lithium (n = 366, p < 0.001) (Supplementary Table 2). As the BD group had higher levels of *RBPJ* mRNA we also controlled for diagnosis. Our result remained significant after controlling for diagnosis (*t* = 3.35 *p* = 0.001 *F* = 5.63 *n* = 400) and when investigating lithium in the BD group alone (*t* = 3.51 *p* = 0.001 *F* = 3.42 *n* = 148). This association does not seem to be dosage dependent, as we observed no correlation between serum levels of lithium and *RBPJ* expression. We found nominally significant effects of antipsychotics and mood stabilizers on DLK1 levels, and a weak dose dependent association between antidepressants and DLL1 levels (Supplementary Table 2).

## Discussion

In the present study we evaluated circulating levels of secreted Notch ligands from plasma and mRNA expression of Notch family members and transcripts that may influence Notch signaling in whole blood in a large population of patients with SCZ and BD as well as in heathy controls. We found significant differences in the concerted regulation of Notch mRNA species between patients and controls, in addition to an increase in circulating levels of secreted Notch ligands. Our main findings include (i) raised levels of DLL1, which is a soluble Notch ligand with potential inhibiting effect on Notch signaling, (ii) down-regulation of *PSEN1* and *RBPJ* (SCZ), potentially impairing intracellular Notch signaling, and (iii) up-regulation of *RFNG* mRNA, which may inhibit ligand-receptor interactions, further impairing Notch signaling.

DLL1 and DLK1 are soluble Notch ligands that are cleaved from their respective transmembrane forms by ADAM proteins, and likely inhibit Notch signaling^19,20^. Thus, the increased plasma levels of DLL1 and potentially DLK1 in SCZ and BD could reflect attenuated Notch activity in these disorders. We have previously identified elevated ADAM17 mRNA levels in BD, suggesting that ADAM17 may be involved in the heightened shedding of Notch ligands in our patient group^21^. Fringe proteins modulate Notch signaling by sensitizing notch receptors to delta ligands and attenuating notch-serrate interactions^22^. Radical fringe (RFNG) is abundantly expressed in the rat brain, and has been found to negatively modulate Notch signaling^23^. Both SCZ and BD patients had significantly elevated *RFNG* expression compared to HC. This further supports a possible impediment in Notch signaling in patients *vs*. controls. PSEN1- controlled γ-secretase activity is essential for Notch signaling as it releases the Notch intracellular domain (NICD) that subsequently enters the nucleus to regulate gene activity. Thus, decreased *PSEN1* expression in patients also favors attenuated Notch signaling, but may also have multiple functions outside of the γ-secretase complex^24^. The implications of enhanced expression of *DTX3L* are less clear. DTX3L belongs to the deltex family, which can facilitate intracellular trafficking of the NICD from the membrane to the nucleus. However, several proteins interact with deltex, and together they destabilize Notch receptors, acting as Notch signaling inhibitors^25^.

In the nucleus, the RBP-J protein enables NICD to bind to DNA and regulate the expression of notch target genes (*HES* and *HEY*). SCZ patients had decreased *RBPJ* mRNA expression compared to HC. *RBPJ* deletion in mouse embryonic brain causes neural stem/progenitor cells to prematurely differentiate into neurons thus depleting the neuronal stem cell population^26^. There is also evidence indicating that RBP-J is necessary for hippocampal-dependent learning and memory in mice^27^. Thus, a shift towards downregulation could hamper proliferation in the brain and interfere with hippocampal functions. The downregulated co-repressors (*i.e. CTBP*s and *HDAC*s) and upregulated co-activator *KAT2B* could imply a shift in favor of increased target gene transcription especially in SCZ *vs* controls. However, as the mRNA expression of *RBPJ* is downregulated, the significance of these alterations is unclear, possibly reflecting compensatory mechanisms. Furthermore, these transcriptional regulators are not restricted to Notch signaling and could be related to other signaling pathways^28^. In contrast to our finding of decreased *HDAC1/2* in SCZ, a recent smaller study demonstrated increased *HDAC1* mRNA in blood leukocytes and in the hippocampus and prefrontal cortex of patients with SCZ who were subjected to early life stress^29^.

Activation of the Notch signaling pathway induces transcription of target genes (*HES* and *HEY*). Hes and hey proteins predominantly act as transcriptional repressors^30^ and, depending on cellular context^31^, impede cell differentiation, and prompt cell proliferation also in the adult brain^26^. We did not observe altered expression of target genes in this study, thus our finding of an attenuated pattern of Notch signaling pathway should be interpreted with some caution.

We show for the first time in a naturalistic study that patients taking lithium had higher levels of *RBPJ* expression compared to patients not medicated with lithium but using other psychotropic medication. This finding is in line with a previous study that proposed that lithium may activate the Notch pathway through the inhibition of glycogen synthase kinase-3β^32^.

Our finding of a possibly hampered Notch signaling may seem at odds with the enhanced T cell response and chronic low grade systemic inflammation in SCZ and BD. However, the role of the adaptive immune system and T cell-mediated immune responses in these patients are still unclear, with some studies favoring a TH2 shift at least in SCZ^33,34^. Notch has emerged as an important regulator of TH-cell differentiation^35^ and stimulation with Notch ligands, including DLL1, promotes a TH1 phenotype which may be inhibited by soluble DLL1^36^. Thus, our finding of increased plasma DLL1 and hampered Notch signaling as reflected by decreased *RBPJ*, is compatible with an altered T cell function potentially favoring development of Th2 cells. A recent meta-analysis suggested a shift towards a TH2 phenotype in SCZ based on circulating levels of typical TH1 and TH2 cytokines while *in vitro* studies favored a TH1 response in the patients. Thus, the role of Notch on T-cell mediated immune responses in the psychiatric disorders needs further study.

There are some limitations to the current study. Firstly, using whole blood as proxy for the brain has limitations, especially when we take into consideration that the Notch signaling pathway is almost exclusively dependent on cell-cell interaction. However, current technology does not yet enable us to investigate gene expression *in vivo* in the brain. We are thus dependent on either post-mortem studies which are limited by artificial changes in gene expression, or alternatively, evaluation of easily accessible tissues that may reflect processes in the brain to a certain degree^37^. Secondly, the use of whole blood does not necessarily reflect the levels in various leukocyte subsets such as T cells and monocytes. Third, due to the naturalistic nature of our study most patients were using a combination of psychotropic medication which prevented exploration of medication in monotherapy. Fourth, the patients were not completely matched with controls in relation to age, ethnicity and sex, but these factors were adjusted for in the statistical analyses.

In conclusion, we demonstrate altered Notch signaling in whole blood in SCZ compared to HC. Although less clear, our results also indicate altered Notch signaling in BD compared to HC. Further, we show an association between the use of lithium and Notch pathway activation. The demonstrated pattern of Notch molecules suggest that the Notch signaling pathway may be compromised in patients compared to controls. There is emerging evidence from rodent studies that Notch signaling is important in adult brain, and partakes in the regulation of adult neural stem cell migration, morphology, synaptic plasticity and survival of neurons^8^. Future studies should be aimed at further exploring the Notch signaling pathway in severe mental disorders, and investigate if therapy targeting this system could be relevant in psychotic disorders.

## Methods

The present study is part of the Norwegian Centre for Mental Disorders Research, University of Oslo and Oslo University Hospital, and collaborating Norwegian hospitals^13^. The study is approved by the Regional Committee for Medical Research Ethics and the Norwegian Data Inspectorate and all research was performed in accordance with these guidelines and regulations. We obtained written informed consent from all participants. The biobank is approved by the Norwegian Directorate of Health.

### Participants

We included patients in the study if they had DSM-IV diagnoses of schizophrenia spectrum disorders or bipolar spectrum disorders, IQ >70, and age between 18 and 65 years (for details see ref.^13^). We randomly selected healthy controls without a prior history of severe psychiatric disorders (or in any of their first-degree relatives), or substance/alcohol abuse/dependency from the same catchment area from the National Population Registry (www.ssb.no) (for details see ref.^13^). For the present analyses, we included patients and controls if they had no coexisting autoimmune or inflammatory disease, cancer or ongoing infections, were not using anti-inflammatory drugs, and had C-reactive Protein (CRP) levels below 20 mg/L.

#### Clinical Assessments

The clinical assessment and diagnostic interviews were carried out by a team of psychologists and physicians who were trained until satisfactory inter-rater reliability was obtained^38,39^. We used the Structured Clinical Interview for DSM-IV Axis I Disorders^40^ to obtain diagnoses. We evaluated clinical symptoms using the Young Mania Rating Scale (YMRS)^41^, Inventory of Depressive Symptoms (IDS)^42^, Calgary Depression Scale for Schizophrenia (CDSS)^43^ and Positive and Negative Syndrome Scale (PANSS)^44^. Global functioning and symptom load was measured with the Global Assessment of Functioning (GAF), split version. The scale has two measures reflecting functioning (GAF-F) and symptom load (GAF-S)^45^.

### Plasma protein assessment

DLL1 and DLK1 were measured in duplicate using commercially available antibodies (R&D Systems, Abingdon, UK) in a 384 format using a combination of a SELMA (Jena, Germany) pipetting robot and a BioTek (Winooski, VT, USA) dispenser/washer. Absorption was read at 450 nm with wavelength correction set to 540 nm using an ELISA plate reader (Bio-Rad, Hercules, CA, USA). Intra- and inter-assay coefficients of variation were 3.9% and 3.7% for DLL1 and 5.0% and 8.4% for DLK1, respectively. We observed no diurnal variation for DLL1 and DLK1: in a smaller group of HC (n = 13) blood was collected at 4 time-points within 24 hours (mean intra-individual CV ± SD was 5.8 ± 5.2%, *p* = 0.27 for DLL1; and CV ± SD = 5.8 ± 6.4%, *p* = 0.86 for DLK1). Further, we found no postprandial variation for DLL1 and DLK1 (n = 13, fasting vs. non-fasting; mean intra-individual CV ± SD was 11.1 ± 7.6%, *p* = 0.15 for DLL1 and CV ± SD = 9.8 ± 11.0%, *p* = 0.84 for DLK1). Detection limits were 10 pg/mL and 25 pg/mL for DLL1 and DLK1, respectively, as defined as 3xSD of assay buffer (n = 10).

### RNA isolation and microarray analysis

#### RNA isolation

Blood samples were collected using Tempus Blood RNA Tubes. Total RNA was extracted with ABI PRISM 6100 Nucleic Acid PrepStation and TEMPUS 12-port RNA Isolation Kit according to manufacturer’s protocol. High-Capacity cDNA Reverse Transcription Kit was used for reverse transcription of 1 µg RNA.

#### Global Transcriptomics Analyses

We selected 49 Notch pathway related genes using the Kyoto Encyclopedia of Genes and Genomes database (http://www.genome.jp/kegg/pathway.html, hsa04330, version date 5/9/17). In addition, we included *HEY1* as it is a well-recognized Notch signaling pathway target gene^30^. For each sample 200 ng of total RNA was biotin labelled and amplified using the Illumina TotalPrep-96 RNA Amplification Kit (Thermo Fisher, Waltham, MA, USA). Global analysis of gene expression was performed with Illumina HumanHT-12 v4 Bead Chip (Illumina, San Diego, CA, USA) consisting of more than 47 000 probes (ie. transcripts). For this purpose, 842 samples (263 HC, 338 SCZ and 241 BD) passed labeling and scanning. Raw microarray scan files were exported using the Illumina GenomeStudio software and loaded into R for downstream analysis using specific packages provided by BioConductor^46^. Lumi was used to detect outliers^47^. ComBat from the SVA R package was used to correct for technical batch effects, like RNA extraction batch, RNA extraction method, DNase treatment batch, cRNA labelling batch and chip hybridization^48^. Further quality control, quantile-normalization and log2-transformation were done using Limma^49^.

### Association between medication and protein/mRNA

We used the defined daily dose (DDD) of psychotropic medications, which is the assumed average maintenance dose per day for a drug used for its main indication in adults, to investigate associations between medication and proteins/mRNA. We calculated the dose relative to DDD for antipsychotics, mood stabilizers and antidepressants according to the guidelines from the World Health Organization Collaborating Center for Drug Statistics Methodology (https://www.whocc.no/atcdd), and used serum concentration levels for lithium. We selected key Notch signaling pathway related genes whose expression was significantly altered in our analyses (*PSEN1*, *RBPJ* and *RFNG*) in addition to plasma proteins.

### Statistical analysis

#### Plasma proteins

We used the SPSS software package for Windows, version 24.0 for the statistical analyses of demographic data and plasma proteins. We assessed data normality using the Kolmogorov-Smirnov and Shapiro-Wilk tests. As distributions were skewed, we used the Kruskal-Wallis test, the Mann-Whitney U test and the chi-square test to investigate differences in demographic data and plasma proteins between groups. Associations between medication and proteins/mRNA were investigated by Spearman’s Rank correlation test. We controlled for age and sex in linear regression models. We inspected the histogram and the normal P-P plot of the regression standard residuals. In case of skewed distribution of the residuals we repeated our analyses using ln transformation of the dependent variable, and then by removing outliers defined as above −3 and below 3 studentized deleted residuals saved from the regression analyses.

#### Global Transcriptomics

Analysis was performed on batch-adjusted log2-transformed values. We used the R software environment to investigate group differences in mRNA expression. We fitted a linear model using age, sex and *Bmal1* expression as covariates. Bmal1 is a critical component of the molecular clock^50^, and we used its expression level to adjust for differences in time of blood sampling and circadian rhythm between patients and HC.

#### Medication

We used ANCOVA to explore the effect of medication while controlling for age, sex and other medication groups.

#### Correction for multiple testing

We corrected for multiple testing according to the Bonferroni method. Alpha was set at *p* < 0.001 for our main microarray analyses (investigating 49 genes), and at *p* < 0.03 for the two plasma proteins. Our secondary analyses investigating the effects of medication were explorative in nature, and we also applied Bonferroni to correct for these analyses separately from the main analyses. Alpha for the secondary analyses was thus set at *p* < 0.003 (correcting for 20 tests).

### Data availability

The datasets generated and analyzed during the current study are not publicly available due to Institutional Review Board restrictions but are available from the corresponding author on reasonable request.

## Electronic supplementary material

Supplementary information
