# Supplementary material for: Attenuated Notch signaling in schizophrenia and bipolar disorder
Source: Sci Rep. 2018 Mar 28;8:5349. doi: 10.1038/s41598-018-23703-w (PMC5871764; doi:10.1038/s41598-018-23703-w)
Supplement: Supplementary file 1 — Supplementary information [file 41598_2018_23703_MOESM1_ESM.pdf]

Title page

Attenuated Notch signaling in schizophrenia and bipolar disorder

Eva Z. Hoseth MD[1,2], Florian Krull PhD[1], Ingrid Dieset MD PhD[1], Ragni H. Mørch MD[1], Sigrun Hope MD PhD[1,3], Erlend S. Gardsjord[1], Nils Eiel Steen MD PhD[1], Ingrid Melle MD PhD[1], Hans-Richard Brattbakk[5,6], Vidar M. Steen MD PhD[5,6], Pål Aukrust MD PhD[4, 7-9], Srdjan Djurovic PhD[11,12], Ole A. Andreassen MD PhD[1], Thor Ueland PhD[4,8,9,10]*

[1]NORMENT, KG Jebsen Centre for Psychosis Research, Institute of Clinical Medicine, University of Oslo, and Division of Mental Health and Addiction, Oslo University Hospital, Oslo, Norway

[2]Division of Mental Health and Addiction, Møre and Romsdal Health Trust, Kristiansund, Norway

[3]Departent of Neurohabilitation, Division of Neurology, Oslo University Hospital, Oslo, Norway

[4]Research Institute for Internal Medicine, Oslo University Hospital Rikshospitalet, Oslo, Norway

[5]NORMENT, KG Jebsen Centre for Psychosis Research, Department of Clinical Science, University of Bergen, Norway

[6]Dr. Einar Martens Research Group for Biological Psychiatry, Center for Medical Genetics and Molecular Medicine, Haukeland University Hospital, Norway

[7]Section of Clinical Immunology and Infectious Diseases, Oslo University Hospital Rikshospitalet,

[8]Instiute of Clinical Medicine, Oslo University Hospital Rikshospitalet, Oslo, Norway

[9]K.G. Jensen inflammatory Research Center, University of Oslo, Oslo, Norway

[10]K. G. Jebsen Thrombosis Research and Expertise Center, University of Tromsø, Tromsø, Norway

[11]Department of Medical Genetics, Oslo University Hospital, Oslo, Norway

[12]NORMENT, KG Jebsen Centre for Psychosis Research, Department of Clinical Science, University of Bergen, Bergen, Norway

Supplementary Table 1. Nominally significant differences (0.001 < p < 0.05) between patients and controls in Notch signaling pathway gene mRNA expression after controlling for age and gender.

	Genes	specificity	SCZ vs. HC	BD vs. HC	SCZ vs. BD
			B	*B*	*B*
Receptor and Cytoplasm	*MFNG*	+++	-.05**	-.03*	-.01
	LFNG	+++	.01	.02*	-.02
	RFNG	+++	.02**	.02**	.00
	DTX1	+++	.02*	.01	.02
	NOTCH1	+++	-.04	-.08**	.04
	DTX2	+++	.01	.02*	-.02
	DTX2	+++	-.04*	-.03	-.01
	PSEN1	++	-.03**	-.03**	.00
	PSEN2	++	-.01	-.02*	.01
	APH1B	+++	.04**	.03	.01
	DVL1	++	.01	-.01	.02*
	DVL1	++	-.01	.00	-.01*
	DVL2	++	-.02*	-.02	.00
	NCSTN	+++	.00	-.05**	.05**
Nucleus	*CIR*	+++	-.01	.05*	.05*
	CTBP1	++	.01	.03**	-.02
	KAT2A	++	-.04*	.02	-.05**
	HES1	++	.01	.02*	-.01
	NCOR2	+++	.01	.01*	.00
	NCOR2	+++	-.03	.02	-.05**
	RBPJ	+++	-.01	.00	-.01*
	RBPJ	+++	-.01*	-.02**	.01

*p < 0.05 **p < 0.01

Specificity: + (unspecific, involved in many pathways), ++ (involved in up to 3 additional pathways *e.g.* Wnt, NF-kappa), +++ (exclusively Notch pathway related gene).

Abbreviations: SCZ=Schizophrenia; BD=Bipolar disorder; HC=Healthy controls; *B*=Unstandardized regression coefficient.

Gene names are listed according to the HUGO Gene Nomenclature Committee. The genes represent probes, and are different from the probes used in Table 1.

Results are given as effect size estimates from the linear regression analysis after correction for age, sex and *BMAL1* expression.

Supplementary Table 2. Associations between daily defined dose of medication, Notch ligand levels in plasma and mRNA expression. Group effects of medicated vs. non-medicated patients.

	n		Notch ligands		*n*		mRNA		
			DLL1	DLK1			PSEN1	RBPJ	RFNG
DDD associations:									
Antipsychotics	603		.06	.06	299		.08	.00	.05
Lithium	54		.16	.10	31		.11	-.12	.06
Mood stabilizers	142		.08	.03	101		.05	-.20	-.14
Antidepressants	30		**.15***	-.04	120		.18	.03	-.10
Group effects:	yes (*n*)	no (*n*)			yes (*n*)	no (*n*)			
Antipsychotics	690	124	1.87	**2.08***	348	53	.75	-1.18	.10
Lithium	63	751	-.91	-.36	35	366	-.52	4.46***	-1.57
Mood stabilizers	145	669	-.40	**2.38***	101	300	-.32	1.30	-.33
Antidepressants	248	566	.35	.11	135	266	.51	.94	-.16

*$p < 0.05$ ***$p < 0.001$

Abbreviations: DDD = daily defined dose; DLL1= delta like canonical Notch ligand 1; DLK1= Delta Like Non-Canonical Notch Ligand 1; yes=using the specified medication; no=not using the specified medication. Gene names are listed according to the HUGO Gene Nomenclature Committee.

We used serum concentration of lithium instead of DDD.

Associations and group effects are given as *t* from analyses of covariance with age, gender and other medication groups as covariates. Results are significant if $p<0.003$ after correction for multiple testing.

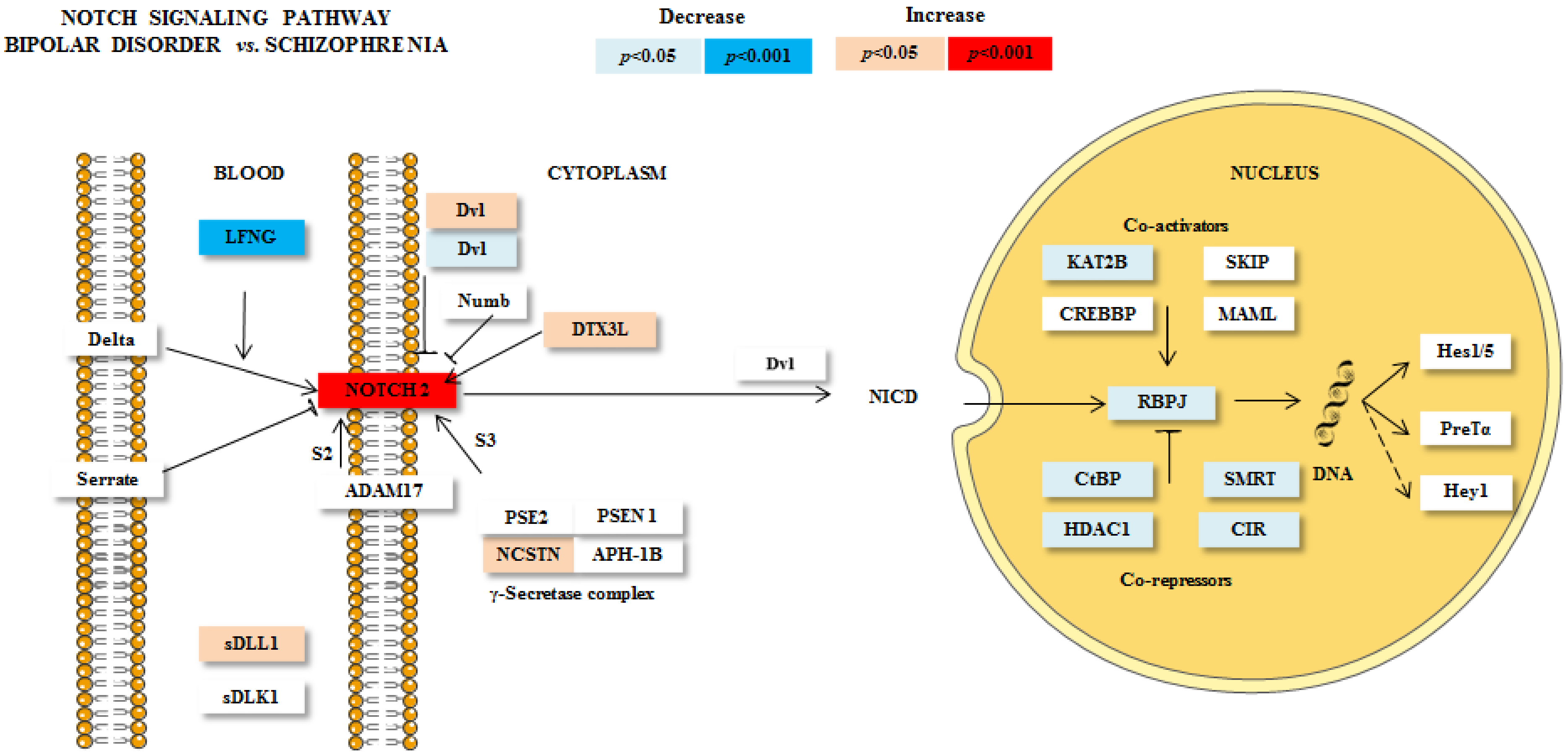

Supplementary Figure 1 Summary of significant and nominally significant findings in Notch pathway mRNA expression between the schizophrenia and the bipolar disorder group after controlling for age, gender and *Bmal1*.
Results are given as *p*-values, adjusted for multiple testing, where significant results are indicated in red/dark blue (for increased/decreased mRNA expression) and nominally significant results (0.001 < p < 0.05) are shown as pink/light blue (for increased/decreased mRNA expression).
